# An integrative computational approach to effectively guide experimental identification of regulatory elements in promoters

**DOI:** 10.1186/1471-2105-13-202

**Published:** 2012-08-16

**Authors:** Igor V Deyneko, Siegfried Weiss, Sara Leschner

**Affiliations:** 1 Molecular Immunology, Helmholtz Centre for Infection Research, Inhoffenstr. 7, 38124, Braunschweig, Germany

**Keywords:** DNA motifs, Cis-regulatory modules, Transcriptional regulation

## Abstract

**Background:**

Transcriptional activity of genes depends on many factors like DNA motifs, conformational characteristics of DNA, melting *etc*. and there are computational approaches for their identification. However, in real applications, the number of predicted, for example, DNA motifs may be considerably large. In cases when various computational programs are applied, systematic experimental knock out of each of the potential elements obviously becomes nonproductive. Hence, one needs an approach that is able to integrate many heterogeneous computational methods and upon that suggest selected regulatory elements for experimental verification.

**Results:**

Here, we present an integrative bioinformatic approach aimed at the discovery of regulatory modules that can be effectively verified experimentally. It is based on combinatorial analysis of known and novel binding motifs, as well as of any other known features of promoters. The goal of this method is the identification of a collection of modules that are specific for an established dataset and at the same time are optimal for experimental verification. The method is particularly effective on small datasets, where most statistical approaches fail. We apply it to promoters that drive tumor-specific gene expression in tumor-colonizing Gram-negative bacteria. The method successfully identified a number of potential modules, which required only a few experiments to be verified. The resulting minimal functional bacterial promoter exhibited high specificity of expression in cancerous tissue.

**Conclusions:**

Experimental analysis of promoter structures guided by bioinformatics has proved to be efficient. The developed computational method is able to include heterogeneous features of promoters and suggest combinatorial modules for experimental testing. Expansibility and robustness of the methodology implemented in the approach ensures good results for a wide range of problems.

## Background

The regulation of gene transcription is accomplished through binding of transcription factors (TFs) to specific regions on DNA called binding sites (BSs or TFBSs), followed by the transmission of regulatory signals to the transcriptional complex. Promoters, that integrate many of such individual signals, provide a particular level of gene transcription, which can be measured experimentally. Deciphering the regulatory logic that forms such integral signals is a challenging reverse engineering problem. Assuming that every gene responds to many different cell situations, the identification of the particular combination of TFBSs (often called *cis*-regulatory module – CRM) responsible for a specific regulatory effect appears to be even more challenging.

Existing computational tools for promoter analysis are focused on the identification of single TFBSs *de novo* or by using known examples, pairwise combinations of TFBSs and clusters see reviews [1-3]. Methods that use known examples of TFBSs, usually require only one set of sequences as input. The accuracy can be adjusted by a threshold parameter. Methods for *de novo* motif discovery search for frequent motifs, estimating their statistical overrepresentation by an internal model and/or using additional negative (background) sequence set. To improve identification, many state-of-the-art methods, both *de novo* and library-based, use additional properties like sequence conservation, genomic localization, correlation with expression, motifs co-occurrence and clustering, functional and structural similarity of bound TFs, surrounding sequence context *etc*. [2,4]. A significant disadvantage of such methods is their reduced applicability due to the need of these data, which are not always available.

Oveall, more than a hundred methods have been proposed for motif discovery to date [1]. Although, these algorithms represent a large variety with respect to search strategies and underlying mathematical models, it is still not possible to identify the best or several best methods since the performance of such methods greatly depends on a particular dataset. For most of the methods, the predictive value lies somewhere between 0.05 and 0.1 [5]. Thus, it provides at best 1 true motif per 10 predictions.

The biological task that led to the current work arose from an investigation of bacterial promoters that are specifically active in a cancerous environment. This is of great interest, since several bacteria like *Salmonella* are able to target and colonize solid tumors and therefore represent a promising tool for anti-cancer therapy. One goal is to use such bacteria as vectors to express therapeutic agents directly in the neoplastic tissue. Therefore, identification and characterization of tumor-specific promoters is a crucial step towards safe and effective medical applications.

A set of 12 promoters of *Salmonella* genes upregulated in a murine cancer model has been identified [6]. A first approach to analyze these promoters using MEME software revealed a motif called *tusp* (*tu*mor *sp*ecific) that was common to most tumor specific promoters [6]. However, further experimental investigations revealed an additional promoter that did not comprise the *tusp* motif. Nevertheless it was tumor specific. MEME and other bioinformatic tools were applied to the extended dataset (12 promoters from [6] and the new one) and several statistically significant single motifs and combinations thereof were found. Each may explain activation in cancer and quiescence in spleen – the tissue, used as an example where no expression should occur. Based on this, we concluded that the hypothesis that expression of tumor specific promoters is regulated solely by the *tusp* motif or by its variants was an oversimplification.

**Figure 1:**
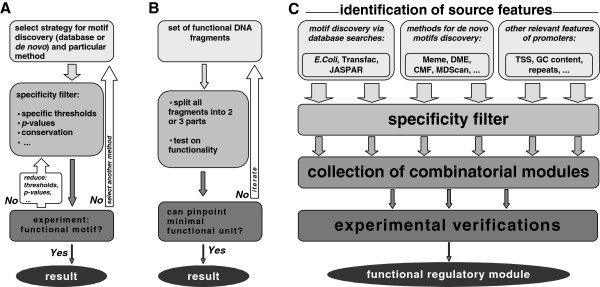
**Workflow diagrams for the experimental discovery of *cis- *regulatory modules. A**) Conventional workflow used to identify regulatory modules in promoters. **B**) Workflow based exclusively on experimental research. **C**) Integrative approach for identification of regulatory modules.

The workflow of the further analysis followed the scheme represented in Figure 1A. By selecting the proper method, thresholds and *p*-values, most significant motifs could be determined and subsequently subjected to experimental verification. By reducing constraints of the method, suboptimal motifs could also be tested. However, during the course of initial assessments of available methods applied to our dataset, we found that each application of a new motif finding program added to the list of potential candidates. Taking into account that the true predictive rate of each method hardly reaches 10% [5], experimental verification of all possible motifs was concluded as unreasonable and the workflow as not effective.

Another motif discovery workflow consists in the experimental verification of all subsequences of a functional regulatory DNA fragment (Figure 1B). By splitting and testing the sequences for functionality, it is straight forward to pinpoint the minimal functional unit [7]. Although this approach guaranties results, it was considered as too laborious and time consuming. In addition, it would fail in case of several motifs that have to interact over a distance (some pros and cons of different workflows can be found in Table 1).

**Table 1:** **Positive and negative characteristics of different workflows shown in Figure**1**for the discovery of functional regulatory units**

**Workflow A**	**Workflow B**	**Workflow C**
+ ease of application	+ very straightforward, no parameters or thresholds	+ can integrate many existing programs
+ software is available	+ guaranteed result in several rounds	+ different algorithms address particular properties of promoters
+ the spectrum of existing methods covers all particular aspects of transcriptional regulation		+ optimization of a collection of combinatorial modules instead of optimization of each module separately
– big number of methods to choose from (over 150 can be found in the Internet)	– may lead to a scission of a functional module rendering all parts non functional	– huge number of predicted features require much memory and CPU = > specificity filtering should be applied before modules optimization
– relative performance of methods differs for different datasets	– high lab work and time investments	
– chance of a correct prediction is ~5-10% [5]		
– impossible to estimate the number of required rounds		

The variety of bioinformatic methods developed to date covers all known aspects of gene transcriptional regulation and obviously should not be neglected in practical applications. As for our problem, since there is no prior indication which principles drive tumor specific expression, many (if not all) methods should be applied. The resulting list of motifs will most probably include true functional one(s), but an experimental testing of all predicted variants would require too many repetitions that are costly and time consuming. Therefore, the bioinformatic problem of motif identification at this point can be reformulated as an identification of a reasonable number of candidate motifs from a longer list of predictions.

Obviously, there is a clear need for a method that is able to integrate many motifs found by any third party program and propose a number of candidates for verification. At best, this output set should be as small as possible, but still should contain all promising variants. This is exactly the concept behind the method presented in this work (see Figure 1C for a schematic overview). In addition to DNA motifs, other features of promoters were included into the analysis and all are further generalized as features. The combinatorial search is based on a genetic algorithm and is robust to the number of features, their positional interdependence, the size of sequence datasets *etc*. Application to the set of tumor specific promoters helped to uncover the shortest functional promoter and to identify regulatory elements comprising it. We could show that this experiment-oriented computational approach has reduced lab time and costs, and has boosted the investigations in the very competitive field of bacteria-mediated cancer treatment [8].

## Results

Analysis of the sequences of 13 tumor specific promoters (see Additional file 1 for graphical representation and Additional file 2 for more detailed information) comprises the following steps: identification of binding motifs for known protein factors, identification of novel motifs, identification of other relevant properties of promoter sequences and a combinatorial analysis of all identified features to build a set of testable hypotheses. Finally, experimental verification of the predicted modules is performed (Figure 1C).

### Known motifs

Regulation of transcription in prokaryotic organisms is mainly studied in *E. coli*. Data collected in DPInteract database [9] was used to construct PWMs. It was suggested to identify an individual threshold for each of 72 constructed PWMs that gives optimal discrimination between positive and negative sets (see Methods). Only 4 PWMs were identified to be specific for positive promoters (listed on in Additional file 1: Figure S1). Setting stricter boundary condition (*C*_PWM_^–^≤0.25) rejected all PWMs. Such a low specificity of the identified motifs can be explained first, by the low number of PWMs in a library and second, by the poor informational content of PWMs, since many are built upon just a few sequences.

### Eukaryotic motifs in prokaryotic genome

To date, the number of known binding motifs for all prokaryotic genomes is orders of magnitude lower than the ones for eukaryotic genomes. TRANSFAC [10] and JASPAR [11] databases comprise tens of thousands of individual binding motifs and over 1500 PWMs. These collections are a highly valuable resource, which in our case can be applied, given that the following is kept in mind. Once identified in a prokaryotic genome, a eukaryotic motif can be bound by a completely different protein factor with different or even opposing cellular function. However, one can also assume that these binding proteins, eukaryotic and prokaryotic, contain similar DNA binding domains. Thereby, the eukaryotic PWM library should be regarded in our case solely as a library of DNA binding domains and their respective motifs. 6 PWMs from the above libraries were identified together with their optimal thresholds (listed in Additional file 1: Figure S1). One motif exhibited improved specificity (*C*_PWM_^–^≤0.25) compared to motifs from the *E.coli* library (Table 2).

**Table 2:** Coverage values for the most specific motif from the respective library in the positive, negative and random datasets

	***E.coli *(4)**^**a**^	**Transfac (6)**	**Meme (6)**	**DME (2)**	**CMF (2)**	**MDScan (5)**	**TSS**	**Poly-(A)**_**8**_
Positive set (*C*^+^)	0.85	0.77	0.77	0.77	0.77	0.85	0.92	0.77
Negative set (*C*^–^)	0.32	0.17	0.07	0.09	0.19	0.11	0.27	0.47
Random set (*C*^*R*^)	0.35	0.19	0.06	0.08	0.21	0.18	0.37	0.59

^a^In brackets are total number of motifs that pass specificity criteria.

### Novel motifs

Several programs for *de novo* motif discovery were tested and the full list is given in the Additional file 1. Derived PWMs were checked for specificity. Only a few programs identified promising motifs, namely: 6 motifs by Meme [12], 2 by DME [13], 2 by CMF [14] and 5 by MDScan [15], some of them being very specific for the positive set (Table 2).

### Other features of promoters

Along with binding motifs, there are other features such as nucleotide content that contribute to regulation of gene expression. Promoters in the positive set are characterized by lower G + C content (0.488±0.0289) than promoters in the negative or random sets (0.532 and 0.509 respectively). Based on this observation, regions longer than 100 bp with a G + C content below 0.401 and above 0.575 (mean plus three sigma) were identified. Interestingly, positive and negative promoters can be discriminated by the presence of AT-rich regions (*C*_AT_^+^ = 0.77, *C*_AT_^–^ = 0.22), but not by GC-rich regions (*C*_GC_^+^ = 0.62, *C*_GC_^–^ = 0.77). This may indicate a transcriptional activity of positive promoters. Thus, promoter fragments comprising such AT-rich regions should be favored for experimental verification.

By visual inspection of the positive promoter set, many poly-A repeats were observed. A search for (A)_8_ with one mismatch revealed a significant overrepresentation of this motif (Table 2). Although the function of such poly-A tracks in promoters remains unclear, this feature was included for further analysis. In addition, all kinds of direct and inverted repeats were identified (see Additional file 1: Figure S1).

Stress-induced duplex destabilization regions (SIDD) were shown to be an important feature of promoters [16]. However, for tumor specific promoters the presence or absence of SIDD regions cannot be regarded as a good discriminator (*C*_SIDD_^+^ = 0.5, *C*_SIDD_^–^ = 0.41). On the other hand, duplex destabilization regions found in positive promoters were characterized by a very dense range of SIDD values with σ=0.003, while for the negative set it was 0.22. This can be interpreted that certain constraints on DNA stability exist in functional promoters. Non functional sequences contain regions of extremely loose DNA together with extremely stable ones (see Additional file 1: Figure S1).

Last, we investigated whether sequences in the positive set exhibit general properties of bacterial promoters. Since promoter recognition tools predict positions of potential transcription start sites (TSS), we will further refer to this feature as TSS. Programs based on sequence alignment kernel [17] and on Hidden Markov Model [18] were applied. The number of promoters with predicted TSSs by the kernel method in the positive set is much higher than that in the negative set (*C*_TSS_^+^ = 0.92, *C*_TSS_^–^ = 0.27, Table 2). Hidden Markov Model prediction shows lower specificity (*C*_TSS_^+^ = 0.83, *C*_TSS_^–^ = 0.43). The characteristic feature of the latter method consists in matching “important” elements, like −10 and −35 boxes and disregarding “unimportant” spacers. In contrast, the kernel method compares the entire sequence of promoters. It is clear that features additional to two known boxes add to specificity of predictions. Thus, predictions by the kernel method were included into further analysis.

### Generating testable hypotheses

Having identified a list of all reasonable features, there is a need to find one or several combinations of such features that are most specific for the dataset. At the same time, it is desired that these combinations locate compactly on the sequences. Unfortunately, there is no reasonable measure of compactness for *cis*-regulatory modules. Application of fixed length windows may either reject true or allow many false hits, if chosen inappropriately. To overcome this problem, we decided to search for modules that locate separately on the sequences. This would also allow straightforward experimental verification of candidate modules by cutting corresponding sequence regions.

Mathematically, this problem could be described as follows: find *K* modules *M*_*i*_ each comprising features (*ϕ*_*1*_*, 0*_*2*_*,…,*$\phi_{n_{i}}$)_*i*_, 1*≤i≤K* such that a) each module covers the biggest possible subset of positive sequences; b) each module covers the smallest possible subset of negative sequences; and c) overall module overlap is minimal. Formally this can be expressed as a set of *K* + 1 equations:

(1) $$\left\{ \begin{array}{l} C_{\left(\varphi_{1},\varphi_{2},\ldots \varphi_{n_{i}}\right)}^{+}/C_{\left(\varphi_{1},\varphi_{2},\ldots \varphi_{n_{i}}\right)}^{-}\to \max,i=1,\ldots ,K\\ \sum_{1\leq i,j\leq K,i\neq j}\left|M\left(\Phi_{i},+\right)\cap M\left(\Phi_{j},+\right)\right|\to \min \end{array}\right.$$

The last condition also ensures that the output modules are not slight variations of each other. This condition on overlap of modules can be regarded as even more important than module coverage, since it directly relates to the number of required experimental steps. The problem formulated in such a general definition cannot be directly solved analytically or computationally. Random walk techniques like genetic algorithm (GA) have proved to be effective in such cases [19].

As initial features, the GA implemented in this work takes known and novel motifs identified in positive and negative sequence sets with optimal thresholds minus 5% to ensure that weaker motifs are included. In addition, this guarantees that each sequence from the positive set contains at least one motif of each type. Repeats, SIDD regions, TSS and regions with high GC content are taken as defined above. Although, some of the motifs appeared to be very similar to each other (for example, MDScan motifs 2, 3 and 5, Additional file 1: Figure S1), all of them were used for combinatorial analysis. In total, 32 types of features (different motifs, TSS *etc.*) that are represented by 1015 instances on 13 sequences were used in combinatorial optimization by GA (see methods).

After 4 independent runs with parameter *K* (number of modules to find) set to five, 20 modules were identified. Excluding identical and grouping similar modules using “OR” logic, 11 candidate modules were manually compiled (Table 3 and Additional file 1: Figure S2). Modules 3, 4 and 6 comprise a pair of motifs FNR and NagC, that was consistently found in each run. For this reason, these modules were left separate. Interestingly, not all of the modules include TSS as a feature. This may look biologically incorrect, since initiation of transcription obviously requires core promoter elements. However, we speculate that in such cases a TSS was probably not recognized by the prediction program or there is a distant TSS that is used. Taking this as an assumption, modules without predicted TSS should be tested like the others.

**Table 3:** Results of the experimental verification of predicted modules

**Predicted *cis *-regulatory modules**		**Fragments used to test the respective module **^**b**^	
		**Experimental round I**	**Experimental round II**
1	Meme1^a^ + TSS	301_1 (+), 48_1 (+)	134_1 (−), 156_4 (−), 212_2 (−)
2	NagC + BRCZ4 + TSS	272_1 (−)	134_1 (−), 134_2 (−), 212_2 (−)
3	(FNR + NagC) OR (FNR + NagC + TSS)	48_2 (−), 156_1 (−), 272_1 (−)	156_4 (−), 134_1 (−), 134_2 (−)
4	(TGIF + FNR + NagC) OR (TGIF + FNR + NagC + TSS)	301_1 (+), 48_1 (+)	134_3 (+), 212_1 (+)
5	Meme1^a^ + HNF1	301_1 (+), 48_1 (+)	134_1 (−), 134_2
6	Meme1^a^ + FNR + NagC	301_1 (+), 48_1 (+)	134_1 (−), 134_2 (−), 156_4 (−)
7	MEF2 + TGIF	301_2 (−)	
8	BRCZ4 + HNF1	271_1 (−)	134_1 (−), 134_2 (−)
9	(RcsAB + MDScan3) OR (RcsAB + MDScan2)	48_2 (−)	134_1 (−), 134_2 (−)
10	(DME1 + RcsAB + Poly-(A)_8_ + TSS)	301_1 (+)	156_4 (−)
11	MEF2 + Meme4	156_2 (−), 272_2 (−), 48_2 (−)	

^a^ Motif identical to *tusp*[6].

^b^ Fragments showing specific expression (+) support the respective regulatory module, fragments showing no- or unspecific expression (−) reject respective modules. After two experimental rounds module 4 is proved to be functional.

### Experimental stage

Identified regulatory modules still exhibit high overlap particularly for some sequences. Therefore, it was decided to carry out experimental verification in two rounds. First, to test longer DNA stretches to eliminate solitary located modules and second, to test shorter sequences to pinpoint the correct module. Following this strategy, inserts 48, 156, 272 and 301 (values represent internal numbering) were selected for the first round as they comprise solitary located modules on their ends. Each insert was split into 3 parts (further indicated by _1, _2 and _3 after the insert number, 3′-5′ orientation), such that the first and the third fragment contains only one module. For testing, sequences covering the second, third and both second and third fragments together were taken. Such a combination of overlapping sequences allows first, to eliminate non functional modules and second, guaranties that the correct motif is still within one of the fragments and intact.

Results of the first round are presented in Table 3 and graphically in Additional file 1: Figure S1. All fragments that cover the 5′ ends of the original inserts showed no regulatory potential indicating that respective modules are not functional (for example, modules 3 and 11 on sequences 156_1 and 156_2 respectively). Two clones 272_1 and 272_3 showed expression both in tumor and spleen. This may indicate that one of modules 2, 3 or 8 is able to initiate transcription but the element responsible for repression in spleen is missing. Altogether, results of the first experimental round showed that modules 2, 3, 7, 8, 9 and 11 should be excluded (Table 3).

For the second round, fragments of length ~50 bp and ~100 bp (exact length varies depending on the underlying motifs) were cloned from sequences 134, 212 and 156. Additionally, a fragment of 165 bp covering the 5′ end of insert 134 was cloned. Results of the flow cytometric analysis of tumor and spleen resident bacteria showed that, for example, none of 156 fragments exhibit transcriptional activity, thus eliminating modules 1 and 10 (Table 3).

The remaining 5 fragments unambiguously indicated the only functional regulatory module – module number 4. This module consists of binding sites for factors TGIF, FNR and NagC and according to predictions requires an optional TSS. Indeed, looking at fragments 212_1, 212_2, 134_1, 134_2, 134_3 and 301_2 (Additional file 1: Figure S1), it is clear, that specific transcription is observed only when all three binding sites are present. Deletion, for example, of the TGIF site leads to an expression arrest (fragments 48_2, 156_1, 272_1156_4 and 134_2) and in one case to unspecific expression (fragment 272_1).

**Figure 2:**
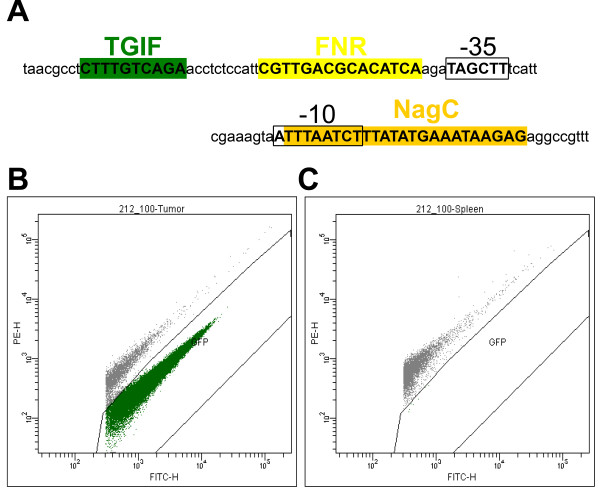
**Tumor specific regulatory module on the sequence of fragment 212_1.** Schematic representation of the regulatory module on the DNA sequence of the fragment 212_1 (**A**). Expression of the GFP_OVA gene under control of the fragment 212_1 in tumor (**B**) and spleen (**C**).

Variation of module 4 without TSS is realized only once, in the sequence 154 where no TSS is found. Obviously, a transcription start on the sequence 154 exists since expression is observed [6]. In this particular case, the promoter finding program might simply have not recognized this site which might be due to an excessively high threshold. Based on this, we state: i) that module 4 should contain four obligatory elements, including a TSS, and ii) that this module specifically defines bacterial gene expression restricted to tumor tissue (Figure 2).

Having identified the structure of the regulatory module responsible for tumor specific expression, we can speculate about functional properties of the predicted single elements. In general, factors FNR and TGIF may both activate or repress transcription [20,21], while NagC is a pure repressor [22]. FNR is also known to be active in tissues suffering from oxygen deficiency. This happens in fast growing tumors, where blood supply is limited. Frequent positional overlap of the NagC motif and TSS may suggest repressive activity of NagC via mechanisms of competitive binding. Unspecific expression of clones 272_1 and 272_3, that lack the TGIF motif and have a non-overlapping NagC motif and TSS may indicate a repressive role of TGIF, which additionally to NagC provides specificity of expression.

On the other hand, it is highly unlikely that eukaryotic proteins exert regulatory function in the prokaryotic cell. Hence, we searched the *Salmonella* genome for a protein, which shows similarity to the TGIF homeobox binding domain [23]. One match showed the longest run of 5 consecutive identical amino acids and an overall similarity of 50%. Surprisingly, this match located in a gene and was in frame with its coding part. The product of this gene is annotated as “putative NADPH-dependent glutamate synthase beta chain or related oxidoreductase” and might be involved in glutamate and nitrogen metabolism. DNA binding activity of this protein is unknown, but extrapolating data reported by Melhuish *et al*., one could speculate on the negative regulatory activity of the identified TGIF-like protein.

Altogether, we have identified the structure of the *cis*-regulatory module responsible for tumor specific bacterial gene expression and suggested individual contributions of its elements.

## Discussion

Experimental identification of gene regulatory modules complemented by computational approaches proved to be efficacious [24]. Depending on the source data and objectives many different computational programs have been developed [1,2]. The novel method presented here, primarily targets the niche where limited data is available. The method is not focused on identification of top scoring hit(s), instead it proposes several meaningful hypotheses that can be efficiently tested. Arranging hypotheses by *p*-values, informational content *etc*., is intentionally not implemented, since limited knowledge will obviously lead to artificial significances, biases and hence will be misleading.

Combinatorial integration of many features from different sources is an essential characteristic of the presented approach. Unlike other methods, any features like gene transcription starts, repeats, GC content in addition to DNA motifs can be included. The method is not sensitive to positionally correlated features since it searches not for individual modules but for an assembly of modules that have minimal mutual overlap on investigated sequences. In contrast to other methods, correlated features should not be grouped [25] or removed [26] from combinatorial analysis. Moreover, this method benefits from the inclusion of such features (and in particular DNA motifs), even when they are extremely similar. This increases a variety of initial seeds in GA, which allows better matching of the goal function. It is very exciting, that the overall true discovery rate does not decrease after the inclusion of so many methods. Indeed, four functional features in each of the 13 sequences divided by the overall number of 1015 identified potential features gives us 0.051, that fully agrees with ~5% true discovery rate reported for most of the methods for DNA motif discovery [5].

Application of many motif discovery methods to the same dataset of tumor specific promoters, showed that the correlation between *de novo* discovered motifs is not as extreme as it should be expected *a priori*. Although, all statistical methods are aimed at the discovery of conserved and overrepresented motifs, correlation is mostly observed between motifs found by the same program but not between different programs (examples are motifs 2, 3 and 5 found by MDScan). This observation additionally supports an inclusion of many methods which in turn increases the chance that the true motif(s) would be in a list of potential candidates.

An interesting point of discussion is the prediction results of methods for *de novo* motif discovery. None of the identified novel motifs has been proved to be functional. There is even no similarity between any of the novel motifs and TGIF, FNR and NagC, as revealed by a motif comparison tool [27]. The closest pair, MDScan8 and FNR, shows similarity of 0.563 which is typical for random matches (for example, similarity between MDScan2, MDScan3, and MDScan5 is > 0.977). This indicates that the usefulness even of the very limited experimentally derived data on binding motifs collected in databases should not be underestimated.

Optimization of compactness of regulatory modules is another significant feature of the method. This additional goal function (1b) greatly influences the performance. As can be seen from Table 4, identified candidate modules do not exhibit superior statistics. From 14 features (13 motifs and TSS) used in modules 1–11 (Table 3), it is possible to combine a “better” module Meme1 + Meme4 + TGIF (“Add1” in Table 4), which is much more specific to the positive promoters (values C^+^ and C^–^). If all 32 significant features identified in this work would be considered, combinations unique to the positive promoter set can be found, e.g. Meme1 + Meme2 + Meme5 (“Add2” in Table 4). Statistical significance of the latter combination would be extreme. In case of optimization exclusively based on *p*-values, the true functional module 4 would rank on place 1685 and most probably would not have been discovered. Positional compactness is the property that helped to reveal the true module. Indeed, the above absolute specific combination occupies on average 306 nucleotides on each sequence, which is almost half of an average insert length of 642 bp in the positive set. Functional module 4 that drives specific expression occupies only 140 bp on average.

**Table 4:** Coverage values of modules on positive, negative and random datasets

**Regulatory module**	**Positive promoter set**		**Negative promoter set**		**Random genomic set**	
	***C***^***+***^	***p*-value**^**a**^	***C***^***–***^	**Rank**^**b**^	***C***^***R***^	**Rank**^**b**^
1	0.92	3.8E-23	0.0181	1	0.0155	1
2	0.92	8.8E-14	0.0776	8	0.0813	7
3	1	2.2E-20	0.0350	3	0.0308	4
4^c^	1	6.5E-19	0.0362	5	0.0399	6
5	1	4.1E-19	0.0371	6	0.0385	5
6	1	3.4E-23	0.0360	4	0.0187	2
7	1	3.5E-14	0.1101	10	0.0922	9
8	1	1.9E-09	0.2018	11	0.2137	11
9	1	1.6E-13	0.1009	9	0.1035	10
10	0.77	5.9E-19	0.0200	2	0.0223	3
11	1	6.8E-15	0.0642	7	0.0813	8
Add1	1	5.5E-31	0.0091		0.0047	
Add2	1	3.6E-47	0.0000		0.000267^d^	

^a^*p*-values are calculated as probability of 13*C^*+*^ successful hits out of 13 trials, with probability of success in one trial *C*^*R*^.

^b^Rank is according to the ratio *C*^*+*^*/C*^*–*^ and *C*^*+*^*/C*^*R*^ respectively.

^c^Functional module 4 does not rank high according to its *p-*value. One can easily find motifs combinations exhibiting superior statistics (for example, Add1 (Meme1 + Meme4 + TGIF) or Add2 (Meme1 + Meme2 + Meme5).

^d^To calculate this value, another random sequence set was generated by repeating 10 times the procedure of random splitting the Salmonella genome. In total the module can be found on 21 of such sequences.

We may speculate that proximal location of regulatory DNA motifs within a module is supported not only by requirements on physical interactions of bound TFs, but also by the random nature of generation of the promoter trap library. Indeed, the promoter trap library represents a collection of random DNA fragments, generated by ultrasonic shearing the salmonella genome and cloned in front of GFP coding gene [6]. Obviously, that the probability that a random DNA fragment entirely covers a dense module is higher than a sparse module. Therefore, the positive set of promoters should be enriched by dense modules, even in case it is not generally reflected in the genome.

We have simulated the number of tests, which would be necessary if the strategy shown on Figure 1B would have been used. When the sequences were divided by half and tested without any bioinformatics, it would have taken 3 rounds and 78 individual tests to find out 212_1 as the “easiest candidate”, easiest, since it almost fits in the 3′ quarter of 212. This would require four times more laboratory work compared to the newly developed approach. When sequences were split into 3 parts (as it was done in this work), it would require much more experiments, since 212_1 would have been divided between sites TGIF and FNR thus rendering all parts nonfunctional. This is also a most probable reason why all 5 fragments of insert 156 showed no expression.

The computational method presented in this work can be easily applied to the analysis of any functional sequences. The key point is an identification of one or many features relevant for the dataset under study. Constraints on the size of a module via optimization of an overlap, is a true benefit compared to methods based on a predefined window. First, it allows exceptions and second, it does not require additional unknown parameter.

Inclusion of optional features, as for example, DNA physico-chemical parameters [28] into the module structure is a promising future advancement of the method. This may help to explain the observed differences in expression levels of tumor specific promoters by the presence or absence of non obligatory features. It may also help to design a new promoter with even increased level of expression by introducing these optional features together with adjustment of core elements. A specific promoter that provides highly restricted expression of therapeutic molecules in tumors has to be the final goal to establish a generally applicable full scale bacteria mediated cancer treatment.

## Conclusions

The integrative computational approach presented here, is designed to speed up experimental investigations of promoters. The method takes into account DNA motifs, repeats, GC-rich regions and any other features of promoters identified by third party programs. Using a combinatorial search and discriminative analysis, the method searches for a collection of combinatorial modules that are highly specific to the given dataset and at the same time are optimal for experimental verification. The method was applied to the analysis of bacterial promoters that show specific activity in murine cancerous tissues. A number of regulatory modules was suggested by the method and the functional module was identified in just a few experimental steps. We show that the method significantly reduced the experimental work required for identification of the functional regulatory module in comparison to either pure experimental or available computer assisted approaches.

## Methods

### Definitions

Let *S* = {*s*_*i*_|*i*≤*N*} be a set of *N =* |*S*| DNA sequences each of length |*s*_*i*_|. *Feature* will be a function that gives a set of sequences, upon it is defined: *ϕ*:*S*→{*ŝ*|∃*i ŝ*⊆*s*_*i*_*ϕ*(*ŝ*) is defined}=*ϕ*(*S*). *Feature coverage* will be a normalized number of sequences in a set that have a subsequence upon a feature is defined: *C*^*S*^_*ϕ*_ =max (*n*|∃*ŝ*_*1*_*,…,ŝ*_*n*_∈*ϕ*(*S*), ∀*i*≠*j*, ∀k *ŝ*_i_⊄s_k_ or *ŝ*_j_⊄s_k_) /|S|. *Feature frequency* will be a *F*^*S*^_*ϕ*_*=*|*ϕ*(*S*)|/Σ|*S*_*i*_|. Similarly we define *module* comprising features *Φ*=(*ϕ*_*1*_*,…,ϕ*_*k*_) as a function *M*:(*Φ,S*)→{(*ŝ*_*1*_*,…,ŝ*_*k*_)|∃*i* ∀*j ŝ*_*j*_⊆*s*_*i*_, *ϕ*_*j*_(*ŝ*_*j*_) is defined}=*M*(*Φ,S*) and *module coverage* as *C*^*S*^_*Φ*_=max (*n*|∃(*ŝ*_*1*_*,…,ŝ*_*k*_)_1_,…,(*ŝ*_*1*_*,…,ŝ*_*k*_)_*n*_∈*M*(*Φ,S*), ∀*i*≠*j*, ∀*k* (*ŝ*_*1*_*,…,ŝ*_*k*_)_*i*_∉*s*_*k*_ or (*ŝ*_*1*_*,…,ŝ*_*k*_)_*j*_∉*s*_*k*_)/|S|.

To define an overlap of two modules we first need to define a module *support sequence*, that is the shortest sequence comprising an entire module *M*(*Φ,S*)^*SuppSeq*^ = min{*ś*| *ŝ*_*1*_⊆*ś,…,ŝ*_*k*_⊆*ś*}. *Modules overlap* will be an overlap of modules support sequences *M*(*Φ*_*1*_*,S*)∩*M*(*Φ*_*2*_*,S*) = {*ś*_*1*_∩*ś*_*2*_|*ś*_*1*_∈*M*(*Φ*_*1*_*,S*)^*SuppSeq*^*, ś*_*2*_∈*M*(*Φ*_*2*_*,S*)^*SuppSeq*^}.

### Datasets

The positive set (“ + ”) contains 12 sequences reported in [6] and one newly found, all showing expression exclusively in tumor; and the negative set (“–”) – 115 sequences, showing no expression (for details see [6]). For a random dataset, the entire Salmonella genome was split randomly into fragments following the same length distribution as in the positive set (average length 640 bp and standard deviation 232 bp), resulting in 7682 sequences.

### Identification of optimal PWM thresholds

Position weight matrices (PWMs) were calculated from position frequency matrices as described in [29]. Programs for *de novo* motif discovery were set (when possible) to provide ≥10 motifs, that were afterwards converted into PWMs. Newly discovered PWMs as well as PWMs from libraries were tested for their specificity to the positive set. For each PWM such a value of threshold was identified, that maximizes the ratio *C*_PWM_^*+*^/*C*_PWM_^*–*^. In addition, the following boundary conditions were set: *C*_PWM_^+^≥0.75, *C*_PWM_^–^≤0.5 and *F*_PWM_^+^/*F*_PWM_^–^≥2. If no value of threshold meets the above criteria, then such PWM is excluded.

### Other features

SIDD regions were calculated as described in [16]. Repeats were found using EMBOSS [30] and classified into GC-rich (G + C≥0.75), GC-poor (≤0.25), and neutral. Poly-A motifs are 8-mers with at most one allowed mismatch. Probabilities of TSS along the sequences were calculated using the demo version of a promoter recognition tool [17]. Threshold 0.7 was identified in the same way as the threshold values for PWMs.

### Genetic algorithm

As an input, the algorithm takes all features with their positions and generates all possible pairs (initial modules). These modules are considered as a population to which the following mutational procedures are iteratively applied: exchange of one feature, add/delete one feature, assembly of two modules into one. After each mutation step, the goal function (1a) is evaluated and the best modules are selected as a population for the next round. The search for minimally overlapping *K* modules (goal function 1b) is implemented using dynamic programming. For computational efficiency, selection of the best *K* modules is done every 10^6^ mutations. If no change in a set of *K* modules is observed in consecutive 100 rounds, the mutation procedure is supposed to be converged to its optimum.

## Experimental protocols

### Construction of different insert fragments

To construct plasmids that contain fragments of the original library inserts [31], oligonucleotides of the desired sequence were either directly ordered (Eurofins MWG Operon, Germany) and cloned into the vector (pMW82) or, for longer sequences, primers were designed accordingly to amplify the fragment from the original plasmid. SL7207 was transformed with plasmids containing the amplification products and plasmid DNA was sequenced to confirm correct sequence of the amplification products.

### Animal experiments

Eight weeks old female BALB/c mice were purchased from Janvier (France) and subcutanteously injected with 5x10^5^ CT26 colon carcinoma cells (ATCC CRL-2638).

When tumors reached volumes of approximately 200 mm^3^, mice were infected intravenously with 5x10^6^ bacteria in 100 μl PBS. 24 hours after infection, respective tissues were removed and homogenized in 2 ml PBS. The homogenates were diluted 1:10 (spleen, liver) or 1:100 (tumors) in 0,1% Triton-X/PBS containing 2 mM EDTA, filtered through a 30 μm CellTrics filter (Partec, Germany) and sorted or analyzed via two color flow cytometry on a FACSAria or LSRII, respectively (Becton Dickinson, USA). Two color flow cytometry is a method that allows distinguishing GFP expressing bacteria from autofluorescent cellular debris since GFP expressing Salmonella have a substantially lower orange/green emission ratio [32]. Additionally, forward and side scatter were used to distinguish Salmonella from larger particles by setting an appropriate scatter gate. For more detailed information see [6]. Procedures involving animals and their care were fully in compliance with the German Animal Welfare Act (Tierschutzgesetz, 1998) and the permission (number 33.9.42502-04-050/09) by LAVES (Niedersaechsisches Landesamt für Verbraucherschutz und Lebensmittelsicherheit).

## Competing interests

The authors declare that they have no competing interests.

## Authors’ contributions

IVD performed bioinformatic analyses of promoters and wrote the manuscript. SW supervised and analyzed experiments and gave critical revision of the manuscript. SL designed, performed and analyzed experiments and wrote the manuscript. All authors read and approved the final manuscript.

## Supplementary Material

Additional file 1 **Supplementary information for the article.** Supplementary file includes lists of programs used for identification of single DNA motifs and regulatory modules, graphical representation of promoters under study (Figure S1) and identified regulatory modules. Click here for file

Additional file 2 Excel file with positions and DNA sequences of all identified features. Click here for file
