# Supplementary material for: An integrative computational approach to effectively guide experimental identification of regulatory elements in promoters
Source: BMC Bioinformatics. 2012 Aug 16;13:202. doi: 10.1186/1471-2105-13-202 (PMC3465240; doi:10.1186/1471-2105-13-202)
Supplement: Additional file 1 — Supplementary information for the article. Supplementary file includes lists of programs used for identification of single DNA motifs and regulatory modules, graphical representation of promoters under study (Figure S1) and identified regulatory modules. [file 1471-2105-13-202-S1.pdf]

Supplementary material for the article: "An integrative computational approach to effectively guide experimental identification of regulatory elements in promoters"

1. The list of programs used for *de novo* motif identification:

All programs were run with default parameters, except number of output modules were set to 10 (when applicable). Programs are taken mainly from review: Sandve, G.K. and Drablos, F. (2006) A survey of motif discovery methods in an integrated framework. *Biol Direct*, 1, 11.

Programs, that identified specific motifs:

DME2 - 2 very specific motifs ($C^{+}_{\mathrm{PWM}} \geq 0.90$; $C^{-}_{\mathrm{PWM}} \leq 0.15$), very convenient output files.
Meme - 6 specific motifs, 3 of them are very specific ($C^{+}_{\mathrm{PWM}} \geq 0.90$; $C^{-}_{\mathrm{PWM}} \leq 0.15$)
CMF - two motifs out of 12 predicted passed specificity criteria.
MDScan - 5 specific motifs, 3 of which are very similar to each other.

Programs, that identified motifs, but they did not pass specificity criteria

LocalMotif
Seeder
Weeder
Scope

Programs, that were not considered for other reasons.

Cbs2- Found on average ~10.5 motifs per sequence. Most are variations of simple repeats like AAAAAA, GGGGGG and so on.
FIRE - identified 2 motifs ACTTT and CCCCGCC, no data is provided to construct PWMs.
MotifVoter - this program selects motifs found independently by a collection of other programs. No results with default options. With option "All programs" selected, the method identified 2 motifs of length ~30, both with just a few conserved positions. The majority of motifs locate on sequences 134, 48 and 301, both motifs are rejected due to low coverage of positive dataset.
AlignACE - all motifs were very unspecific, on average there were 144 motifs per sequence or 3,74 per bp.
MotifSampler - (part of TOUCAN) output are dominated by two similar motifs.

Programs that are not applicable to our dataset.

Improbizer - fails to run with "number of motifs to find" = 6, program reports "sequence data is too big". With "number of motifs to find" =2 program outputs only one motif, than hangs.

Ann-spec 1.0 - searches only in human and yeast promoters.

MotifRegressor - needs expression values. Can not be run just on a set of sequences.

2. The list of programs used for *cis*-regulatory module identification:

All programs were run with default parameters, the list is based on reviews:
1. Klepper K. and Sandve G.K., Abul O., Johansen J. and Drablos F. (2008) Assessment of composite motif discovery methods. *BMC Bioinformatics*, 9, 123.
2. Van Loo P. and Marynen P. "Computational methods for the detection of *cis*-regulatory modules". (2009) *Brief Bioinform*. 10, 509-524.
3. Su J., Teichmann S.A. and Down T.A. (2010) Assessing computational methods of *cis*-regulatory module prediction, *PLoS Comput Biol*, 6, e1001020.

CisModule: found modules of 200bp in length, that have from 1 to 14 instances of 3 different single motifs in all combinations. No general rule for module structure can be established.

ModuleSearcher: This program is a part of TOUCAN project. Results are dominated by 2 frequent motifs (~10 times more frequent than others). CRMs comprising these frequent motifs show good statistical significance, but present not on all sequences.

Stubb: did not identify any hits.

COMET, Cluster-Buster and **Cister:** 3 programs from the same lab that search for dense clusters or sites. All programs treat multiple sequences as one long sequence. Programs found clusters with 6 and more motifs and length > 150bp, most of the clusters overlap adjacent sequences. If sequences are submitted separately, finds no clusters.

Programs not used for other reasons.
ModuleFinder: Only executables are provided. Unclear how use user-defined PWMs.
MCAST: MCAST identifies very long modules. For example, a top hit is a module consisted from 28 binding sites and spanning 620bp on a 1150bp sequence of the insert 48. Though very significant E-value and high score, this result seems to have little practical use.
CMA: Website is partially down (images are not available). No details provided on model parameters (distance, orientation).
ModuleScaner: This program is a part of TOUCAN project. Only scans for CRMs using provided templates.

Not available programs:

HexDiff: Website is not available. No supplementary provided. Google search - No results.
MSCAN: Website does not exist. Email to authors: "The recipient's e-mail address was not found in the recipient's e-mail system". Google search - No results.
CO-Bind: Executables are reported to be on an ftp site. Ftp errors with "no such directory".

EMCMODULE: "Executables upon request", e-mail to authors failed: "user unknown".
HexDiff: link to source code provided in the manuscript is invalid. Google search - no results.
RP - no web site or standalone executables provided.

Supplementary Figure 1S. **Graphical representation of motifs and other features.** Each red/green/grey long arrow represents a DNA fragment. Numbers relate to internal numbering. Sub fragments marked by _1, _2 and _3. Red color indicate expression in tumor but not in spleen, green - no expression and grey - unspecific expression (both in tumor and spleen). For description of individual features see legend below. DNA sequence of individual features can be found in Excel file in supplementary. We may recommend to print the image on A3 or A2 format.

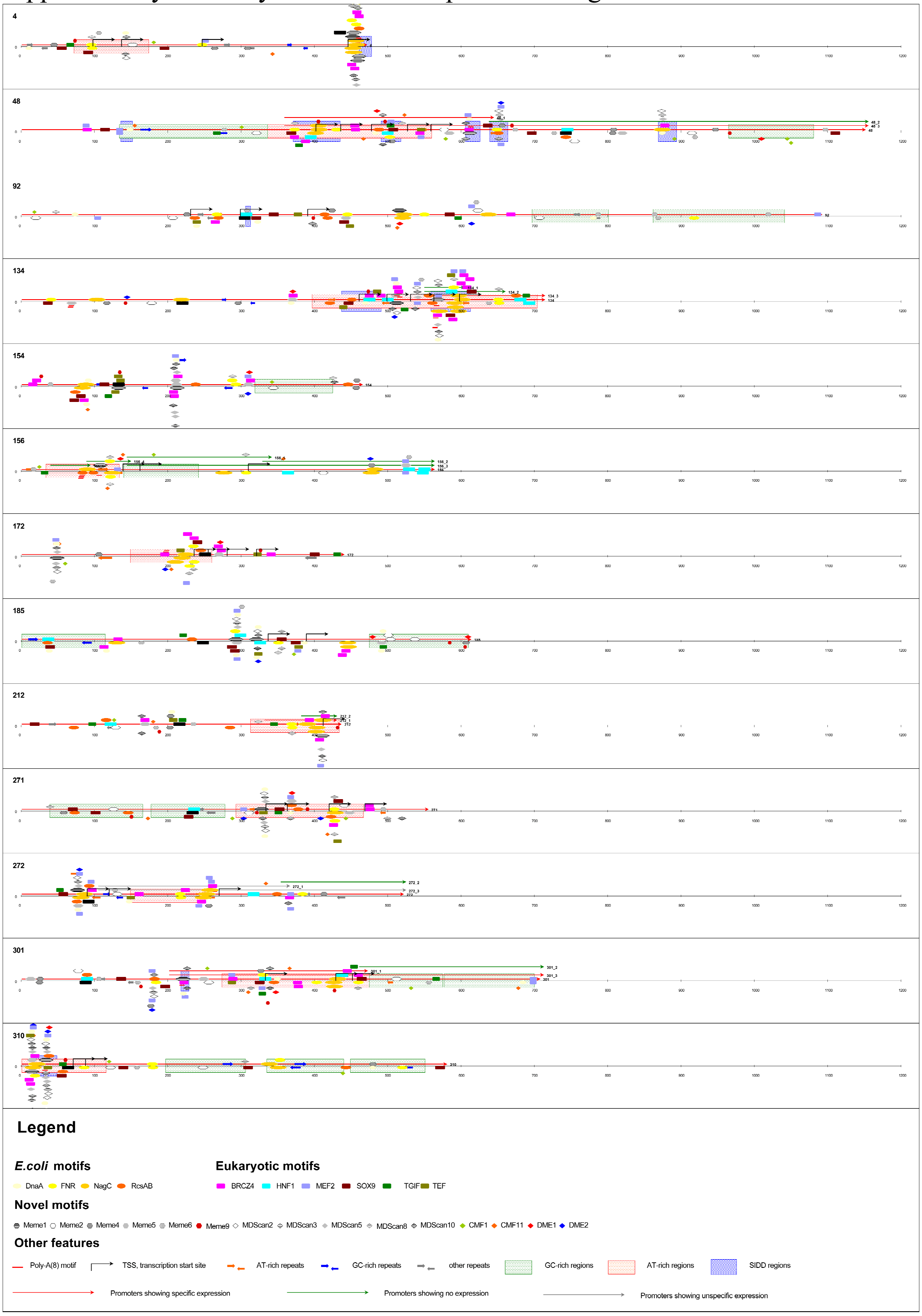

Supplementary Figure 2S. **Graphical representation of promoter modules**

Potential promoter modules responsible for tumor specific transcriptional activation. Similar modules found by genetic algorithm were combined using "OR" logic (modules 3,4,5,9). DNA sequence of individual features can be found in Excel file in supplementary. For description of individual features see legend below.

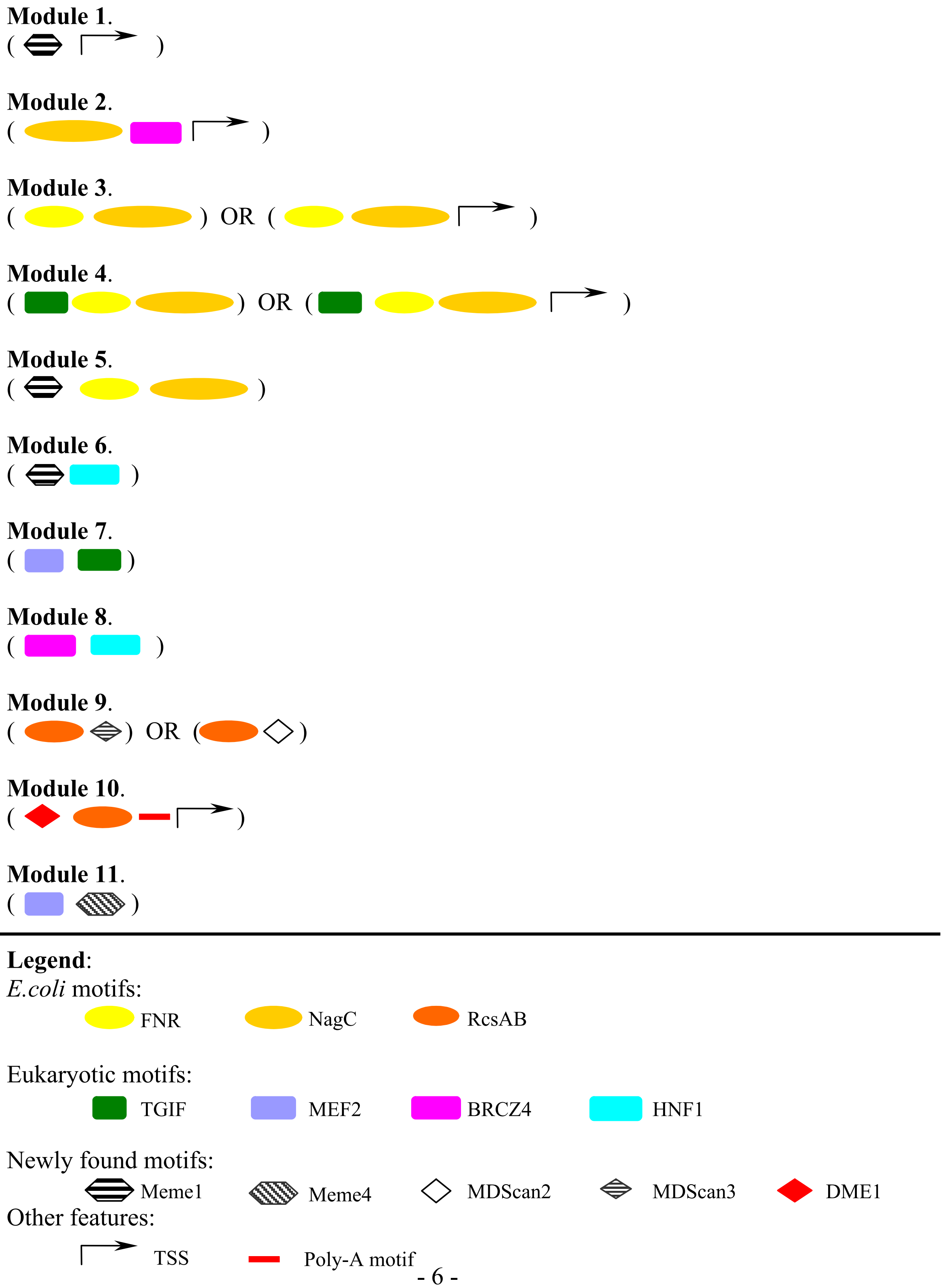

Fasta formated sequences of all tumor specific fragments.

```
>134>Ext_sa_p1_ass.0.358 salm_ass.0.101
gatcccggcgaagcggcccccgtcttaaaggccattgctgaacataagtggatgccggag
gcgatttttttgacgcatcaccatcacgaccatgttggcggagtcaaagagctgttgcaa
cacttcccgcaaatgacggtttatggaccggcggaaacgcaagacaagggagcaacccat
cttgttggcgatggcgatactattcgcgttttaggcgagaaatttactcttttttgccacg
ccgggccacacgttaggacacgtctgttactttagccgcccttacttattctgcggcgac
acgctgttttccggcggctgtggtcgactgtttgaaggcacgccatcacagatgtatcag
tcacttatgaaaattaactctctgcctgacgacacgctcatttgctgcgctcacgaatac
actttagctaacattaagttcgcattgagcatacttccgcacgattcgttcataaatgaa
tattatcgtaaagttaaagagttacgtgtaaaaaaacaaatgacattacccgttattctt
aaaaatgagcgtaagattaatcttttttttaagaactgaagatattgatttaattaacgaa
ataaacaaagaaacaatattgcaacaaccagaagcgcgttttgcatggttaaggtcaaag
aaagacacgttctgataattcttacttgtcattcgctaacttcgccgttatgatcggtcg
tcttttaagcaactattgacacacac
>310>sa_p3_ass.0.258 salm_ass.0.288
gatcgtatcttttttataaaacacaaccattttttataaacatcctgattgaaattgtcat
aaactattacccggagttttggagtccagcaaccaaaggagacggaatgcatcacgctac
cccgcttatcaccaccattgtcggcggccttgtgctcgcttttattctcggcatgattgc
caacaaattgcgtatttctccactggtgggatatctgttagcgggcgttctggcgggacc
ttttaccccgggttttgttgcggataccaaactggcgccggagctggcggagctcggcgt
gattctattgatgttcggcgtcgggctgcattttttcgctgaaggatttgatggcggtaaa
gtctatcgccattcccggcgctgtcgctcagatagcggtggcgacgctgctgggtatggc
gctttccgccgtgctgggatggtcattaatgaccggcatcgtttttgggctctgtctgtc
tacagccagtaccgtcgtcctgctgcgcgcgcttgaggagcgacaactccttgatagcca
gcgcgggcaaatcgccatcggctggctgattgtcgaagatc
>212>sa_p1_ass.0.155 salm_ass.0.63
gatcggttcgctacaggcaatggaggccattaagctactggcgcattacggtcagcctgc
cagcggaaaaatcgtcatgtacgatgcgatgacctgtcagttccgcgaaatgaagttaat
gcgcaaccccggctgtgaggtctgcgggcagtagccactttacggataaacataccaaag
cacggtttatggccgtgctgtatcaacagataattactgtcatcagacgcaaagccagct
tacccaagctccgactgacgatcggctcaagcagcgcggcatcatcttaaacgccatacc
acaacctcaaaccgtgatgttgtacctcatttaacgcctctttgtcagaacctctccatt
cgttgacgcacatcaagatagctttcattcgaaagtaatttaatctttatatgaaataag
agaggccgtttatgatc
>4>first_ass.0.13 salm_ass.0.8
gatctttgatgaacagggacgtctgtgctgttcttcgcgtcttactacagcgattgtgtg
acgttgtcaccataaaagtgtgatgcatattgcttttggtcaaaatgtgcgcagaaaaag
gtgatatactgtgcacgtttacacataagcgaggatgatatgtctactgatttggaccca
acccaactggcgattgaattttttacgccgtgataaaaccgaactttctcccgcgcagtat
ttgaagcgtttaaaacagctggagttagagtttgccgatctcctcaccctctcagcaacc
gaactgaaagaagagatctatttcgcctggcggttgggcgtgcattaataaagtctgtgt
aggccggataaggcgttagccgccatccggctatgttaccaggcaatctcaccagacctt
gtccccaggtaatagataacccccttgattttgtttaaaaaaaatacagatc
>48>sa_p2_ass.0.17 salm_ass.0.120
gatcgatggcttcagcgccaatgcctgccgcttcacaggcgcgaataaaggtggcctgaa
acgcaagatcatcttccggcaacgtaataaacagaccatccgtcggttcgacgcaatggc
gggcgatccgttttaaaatctggttttcactaatgcactcgcgcgcggattccgcgtcgg
taaccgcataacgagcgccgctgtgcagcagcccgtggttacgcccggtcgcgccggtcg
ctatatcatgccgctccaccagaatgacacgtaaaccgcgcagcgcgcagtcgcgggcga
tccctgcgcctgttgctccaccgccaatgataatcacgtcacttgtttgcgagtcgcgag
ttttcattgtttttcctcacagttcgtttttttatcatttagccatacaaatcatatgtaa
tgtttgatttcgcgcataatcgctcactattcgaaaatgaaacgtgatttcgtgcgcctt
tctgaacattagtcataaatctgtaacaatatgtgctgtaattcacattaacgtgacgta
tctttacttaacatcgcggccacactgggagcagcggggttgtttgaacgaactgcggtg
tttacctctaaataaaatatgggccacggaggctacaatatgttgagtatttttaaacca
gcgccgcataaagcgcgcttgccagcggcggagattgatccgacctatcgccgattacgc
tggcagattttcctggggatattctttggctatgccgcgtattatctggtgcgcaagaac
```

tttgccctcgcgatgccctacctggtagaacagggtttttcacgcggcgatctgggcttt
gcgctgtccgggatttccatcgcttatggtttttcgaaatttataatgggttcggtgtcc
gatcgctcgaatccgcgcgtttttctgccggcagggttgattctggccgcagcagtcatg
ttgtttatgggctttgtgccgtgggcgacatccagcatcgccgtgatgtttgtactgttg
ttcctttgcggctggttccaggggatggggtggccgccgtgcggtcgtacgatggttcac
tggtggtcgcagaaagagcgcggcggcattgtgtcggtctggaacggcgcgcataacgtc
ggcggcgggatc
>301>sa_p3_ass.0.148 salm_ass.0.266
gatcgccgcgtgaaaaaccctgttctaccaggtagggcatcgcgagggcaaagttcttgc
gcaccagataatacgcggcatagccaaagaatatccccaggaaaatctgccagcgtaatc
ggcgataggtcggatcaatctccgccgctggcaagcgcgctttatgcggcgctggtttaa
aaatactcaacatattgtagcctccgtggcccatattttatttagaggtaaacaccgcag
ttcgttcaaacaaccccgctgctcccagtgtggccgcgatgttaagtaaagatacgtcac
gttaatgtgaattacagcacatattgttacagatttatgactaatgttcagaaaggcgca
cgaaatcacgtttcattttcgaatagtgagcgattatgcgcgaaatcaaacattacatat
gatttgtatggctaaatgataaaaaacgaactgtgaggaaaaacaatgaaaactcgcgac
tcgcaaacaagtgacgtgattatcattggcggtggagcaacaggcgcagggatcgcccgc
gactgcgcgctgcgcggtttacgtgtcattctggtggagcggcatgatatagcgaccggc
gcgaccgggcgtaaccacgggctgctgcacagcggcgctcgttatgcggttaccgacgcg
gaatccgcgcgcgagtgcattagtgaaaaccagattttaaaacggatc
>154>sa_p2_ass.0.75 salm_ass.0.132
gatccggattacgcaaattaaatgcataaaagccaaaattgcgcgactccgcattcttga
tgagtgaggattgtaatcattgaatttgtgaattaaggtcgccgccgcggagcaatagac
acttagctaatcatataataaggagtttaggatgaaagtcgcagtcctcggcgctgctgg
tggtatcggtcaggcgctggcattacttttaaaaaaccaactgccttcaggttcagaact
ctccctgtacgacatcgctccagtgactcccggtgtggccgttgatttgagccacatccc
caccgctgtaaaaatcaaaggtttctccggtgaagacgcaaccccggcgcttgaaggcgc
tgacgtagtactgatttctgcgggtgtggcgcgtaagccgggtatggaccgttccgacct
gtttaacgttaacgccggcatcgtgaaaaacctggtgcagcagatc
>271>sa_p2_ass.0.51 salm_ass.0.128
gatcgcagcaaacgaaagtggcctcgcctaagctggttaacttcattctgacctgcggca
ccatcggaacaatgctgaccttcgtcgtcaccggcccgattgtagcgcacagcggcccac
aggcggcgttactcaccgcgaatggtctgtatgcggtggtctttgtgatgtgctttgcgc
tcggctttgtatcccgtcatcgtcagcatagcgcgccggctacgcattgataatccttgc
cggatggagacatcgccctccggcactctatccccccctcctgacggggtaggcctgttgg
tctaaaaacccctcatttgtatgttatttgtacaaacctgaaaagcctgacaattccgc
cacttataaaaatccagacaaatcagccatatacccattaagaggtatataaaggtgaat
ttgatttacatcaataagcggggttgctgaatcgttaaggtaggcggtaatagaaaagaa
atcgaggcaaaaatgagcaaagtcagactcgctattatcggtaatggtatggtcggccac
cgctttattgaggatc
>92>sa_p3_ass.0.8 salm_ass.0.215
gatcgctttacaggcgaaccccccttctcaatcgtcatcggatgatttacggtacgttaac
ggcggaactttccacaaccgtacatgcgctggcactgcatacttataccctcaaagagtg
ggaaggattgcaggatactatcttcgcgtcgcctccttgtcgcggcgcgggaagcatcgc
gtagcaaaacggatttgcaactacctccgcttttccagtatgttgctacagaattatgtg
aaaacggcctgcgggccgttttgttttgtctgaattttgagcgtgtcgtacagtattcag
acaaaaattagccgagaattgtgaaaaccgccgcagcatcgcacaatcaccgttctcgac
tcacaaaagtgatgccgctataatgcgccgtcttatatatgaacgtcttcgggatgattc
tgacgacagggaatgtgattgattacgagaacatcccggttccgcgaagcaaatagcacg
tgcttgcggagtagagttgaccgagcactgtgatttttttgaggtaacaagatgcaagttt
cagttgaaaccactcaggggccttggccgccgtgtaacgattacaatcgctgctgacagc
atcgagaccgctgtaaaaagcgagctggtcaacgtagcgaaaaaagtacgtattgacggc
ttccgtaaaggcaaagtaccgatgaatatcgtcgctcagcgttatggcgcttctgttcgc
caggacgtgctgggcgatcattccctggaacgccccgctttcacgctgcctgacgcttat
tgaaagcgtgcaggggcagcagttcagccgttacgtaccggaagacatcaccacgctact
gtcgatgacgcagccgttgaaactgcgcggttttcagccgtgggataccttctgcgatgc
catccatacgatgatgagcaacaccctgctccccgccgacgggaaaggcgttctggtcgc
gctgcgcccggtgccgggcattcgggttgagcaggcgttaacattatgtcggccaaacag
ggccggcgatattatgaccatcggcggcaaccgtctggaaaggtggttatcattctgccg
gggtctaacgatc
>185>sa_p4_ass.0.245 salm_ass.0.365

```
gatcgcgcgctgcccgatgcggaacgacaggaaattattgatatcgtgacgtcatggccg
ggagtcagcggcgcgcacgatctccgcacgcggcagtcagggccgactcgctttattcag
attcatttggaaatggaagataatctgccgctcgttcaggcgcattttgtggctgaccag
gtagagcaggcgattttacagcgttttccgggttcagatgtcattattcatcaggatccc
tgttcagtcgttcccagggaaggcaggaagttcgagcttgtataattgattgttaaaaag
tgagccaggccagcattttgtgtataaattaccgccatttggcctgacctgaatcaattc
agcaggaagggattgttatactatctgtatattcgttggatcgtttcgaagtgcgaaatc
ggcttccggcaatagatttcattttgcattccaaagttcagaggtagtcatgattaagaa
aatcggtgtgttgacaagcggcggtgatgcgccgggcatgaacgcggcaatccgcggtgt
tgtgcgcgcagcgttgacggaagggctggaagtcatgggcatttatgacggctatctggg
cctgtatgaagatc
>172>sa_p4_ass.0.181 salm_ass.0.349
gatcggcaaagaaacgacggatttccgccataatcgccgcacgttttaataaattgggga
tggacgcgctcggctgccaggttgccgtttcgctcatgattctttctccagtttaagaca
aggtcacgaagtctactcgcaacgcgcgggcgaaacaaattttgcgcaggcgtatcgggc
gccttctggagggtaaaaaaagtgatttcagatggtttagtaattaaattaatcaaaatc
aatgataattcatccctctgatacgctaaaaaaatcgaacacgtcaaatttccctcacat
ccctgagactatactgttgtacccataaaggagcagtggaaacgcattcatacttcgcag
aacccagaggctttatctggctgcgcgagggtgaaattacaataatctggaggaatgtcg
tgcaaacctttcaagccgatc
>156>sa_p5_ass.0.121 salm_ass.0.420
gatcgcaaggtgaaaatgagcccaaccctggacaggaagcgttgagcttttcgatgtgcg
ccagttaaaattctggcgtttttttctcaccgaattttctcatttttttctcaacgtgatt
ttcatcactataagaaaatcacgtaagtgcttgaatagtggcggagagagagggattcga
accctcggcggagttaccccccgcaacggttttcgagaccggtccgttcagccgctccggc
atctctccgtatattgcaatgatgccaggtaatttggcattttaacagaccctattcggg
taattttgttcaagtgacgagtttacgagcaaaacgatgattaagtggccctggaaagca
caagaaataacccagaacgaagactggccgtgggatgatgcgctggctatacctcttctg
gtaaacctcaccgcgcaagaacaggctcggcttattgcgctagccgaacgtttttttgcag
cagaaaagactggtagcgctacagggatttgagctcgactcgttaaaaagtgcacgtatt
gcgttaatttttttgcttaccgatc
>272>sa_p6_ass.0.89 salm_ass.0.506
gatcaccaccgataactttgctcgcctgttccatattgacgcgtctcgcctgtcatcaat
ccgttaaacgagtttttttaaagctcgtaattaataaacaaaacgcgtaaagttcaccgc
cacaaaaggggcggtgagcgagcttatggaaacattcggaaactcattttggcagaatgt
gatacttttttaggctatctggcgctgaaacgtgatagccgtcaaacaaaatcagacgtat
ttattttactctgtgtaataaataaaagggcacttagatgtcctgtccacggcggggttc
tccccccctcgccaatgcgtgagaacgtagaaaagcacaaatactcaggagcactctcaat
tatgtttaagaatgcatttgctaacctgcaaaaggtcggtaaatcgctgatgctgccggt
atccgtactccctatcgcaggtatcctgctgggtgtcggttccgctaacttcagctggct
gccagccgttgtatcgcacgttatggcagaagcgggcggatc
```
